# Editorial: New techniques in microbiome research - volume II: Host-microbiome interactions using ‘meta-omics’ techniques

**DOI:** 10.3389/fcimb.2025.1541881

**Published:** 2025-02-05

**Authors:** Tao Lin

**Affiliations:** Department of Molecular Virology and Microbiology, Alkek Center for Metagenomics and Microbiome Research, Baylor College of Medicine, Houston, TX, United States

**Keywords:** microbiome, human health and diseases, new techniques, META-OMICS, metagenomics, metatranscriptomics, metaproteomics, metabolomics

Recent advancements in meta-omics techniques have significantly enhanced our understanding of the microbiome and its association with various human diseases. By integrating data from metagenomics, metatranscriptomics, metaproteomics, metabolomics, and spatial resolution, researchers can comprehensively analyze microbial communities and their interactions with the human host. A central theme of this Research Topic is the role of the microbiome in shaping human health and disease outcomes. Key studies included this Research Topic explore the diversity of microbial species, their functional contributions, and the downstream impacts on human health and diseases.

## Key developments of meta-omics tools in unraveling the mechanisms through which microbiota affect human health and diseases

### Comprehensive microbiome profiling

High-throughput sequencing technologies have enabled detailed characterization of the human microbiome, revealing its complexity and diversity. For instance, the Human Microbiome Project has cataloged thousands of microbial species, providing a foundational understanding of microbial composition in healthy and diseased states (Turnbaugh et al., 2007).

### Integration of multi-omics data

Combining various omics datasets offers a holistic view of microbiome function. Metagenomics provides insights into microbial gene content, metatranscriptomics reveals gene expression patterns, metaproteomics identifies active proteins, and metabolomics quantifies metabolic products. This integrative approach has been pivotal in linking specific microbial functions to human health and disease (Zhang et al., 2019).

### Biomarker discovery and their association with human diseases

Meta-omics analyses have identified correlations between microbiome composition and diseases. Metagenomic analyses have shown that specific microbial taxa and their functional capacities are strongly associated with metabolic health. For instance, certain gut bacteria have been associated with the development of type 2 diabetes (Muller et al., 2021), highlighting potential targets for therapeutic interventions (Fan and Pedersen, 2021). The ability to assess microbial genes and their metabolic outputs through metabolomics has provided new insights into how microbial-derived metabolites, such as short-chain fatty acids, influence human metabolic pathways, offering potential avenues for early diagnosis and targeted therapy.

### Therapeutic interventions

Understanding microbiome-disease associations has led to the exploration of microbiome-based therapies. Probiotic and prebiotic interventions, as well as fecal microbiota transplantation (FMT), are being investigated for their potential to restore healthy microbiome composition and mitigate disease progression. FMT has gained traction as a treatment for recurrent *Clostridioides difficile* (*C. difficile*) infections and has been explored for its potential role in other conditions such as IBD (Ianiro et al., 2021).

### Microbiome and cancer

The impact of the microbiome on cancer has been one of the most intriguing areas of research. Studies suggest that the microbiota can influence cancer progression, immune response, and even the efficacy of cancer therapies. For example, research has shown that microbial species residing in the tumor microenvironment can modulate immune checkpoints and affect the response to immunotherapy. Studies have shown that certain microbial species, such as *Fusobacterium nucleatum*, can exacerbate colorectal cancer by promoting inflammation and genomic instability (Kostic et al., 2013).

### Microbiome and neurological disorders

The gut-brain axis has become a focal point of research into neurodegenerative diseases. Meta-omics approaches have shown that gut dysbiosis can impact neurological health by altering microbial metabolite production, which in turn affects neuroinflammation and brain function (Cryan et al., 2019). This emerging evidence suggests that gut microbiota may play a crucial role in the development of diseases such as Parkinson’s and Alzheimer’s, offering new opportunities for microbiome-based therapies.

### Microbiome and infection

The microbiome profoundly impacts human susceptibility to infections and immune defense. Dysbiosis in human body such as the gut, vaginal, and skin microbiomes is associated with an increased risk of infections (Elkafas et al., 2022). Similarly, dysbiosis in the gut microbiome compromises immune function, leading to a greater risk of gastrointestinal infections, including *C. difficile* infection (Acevedo-Román et al., 2024). Research also highlights the gut-lung axis, showing that gut microbial imbalances can impact respiratory infections, suggesting a systemic role of the microbiome in maintaining immune homeostasis​ (Acevedo-Román et al., 2024). Moreover, sputum microbiota is associated with an severe and critically ill influenza patients (Gu et al., 2023), In addition, evidence indicates that the gut microbiota can alter SARS-CoV-2 virus load and COVID-19 severity (Zuo et al., 2020).

### Microbiome and women health

The microbiome plays a critical role in women’s health, particularly in the context of the vaginal and gut microbiomes. The vaginal microbiome, which is typically dominated by Lactobacillus species, is essential in maintaining a healthy environment by producing lactic acid and maintaining an acidic pH. Disruptions in this balance can result in bacterial vaginosis, yeast infections, and increased susceptibility to sexually transmitted infections (STIs) (Cocomazzi et al., 2023). In cases of bacterial vaginosis and other vaginal dysbiosis, an increase in pathogens like *Gardnerella vaginalis* and *Prevotella* has been linked to adverse reproductive outcomes, such as infertility and miscarriage (Cocomazzi et al., 2023) (Gu et al., 2022).

In addition, oral microbiome changes are associated with the menstrual cycle. Different microbiome profiles were observed during the follicular phase, the early and late luteal phases. Alpha diversity and beta diversity analyses revealed distinct microbial profiles across the four menstrual phases. Probiotic lactobacilli were used in the treatment of vaginal infections (Cohen et al., 2020). The urinary microbiota signatures are associated with different types of urinary diversion.

Furthermore, the gut microbiome also has significant implications for hormonal regulation and immune responses, which can impact conditions like polycystic ovary syndrome (PCOS) and pregnancy outcomes. Studies have shown that alterations in the gut and vaginal microbiota are associated with insulin resistance, hormonal imbalances, and inflammation in women with PCOS, contributing to symptoms like infertility and metabolic disturbances (Cocomazzi et al., 2023) (Gu et al., 2022).

## Key studies featured in this Research Topic

The aim of this Research Topic is to offer a platform for articles that expand our understanding of host-microbiome interactions using ‘meta-omics’ techniques. This Research Topic includes 4 reviews and 14 original research articles, highlighting critical associations between the microbiome and various human diseases. These contributions have made significant strides in advancing our knowledge of the complex interplay between the microbiome and disease states, including the microbiome and cancers, (Cheng et al., Li et al., Cai et al., Zhang et al.); Single-cell analysis and spatial resolution of the gut microbiome (Madhu et al.); Microbiome and Parkinson’s disease (Jia et al.), depression (Li et al.), and insomnia (Wang et al., Li et al.); Oral microbiome changes associated with the menstrual cycle (Yamazaki et al., 2023), vaginal infection (Liu et al.), and urinary diversion (Liu et al.); Microbiome and influenza infection (Gu et al.); Microbiome in *C. difficile* Infection (Vázquez-Cuesta et al.) and split-dose bowel preparations (Zou et al.); Microbiome in fatty liver disease (Niu et al.) and gallstones and cholesterol metabolism (Zhang et al.); and assessing efficacy of clinical disinfectants for pathogenic fungi (Li et al.).

## The microbiome and cancers

A review on respiratory tract microbiota and lung cancer investigates the bacterial communities inhabiting different regions of the respiratory system (Cheng et al.). The emerging evidence suggests that gut microbiota could play a key role in mediating oncogenesis through various mechanisms, potentially opening new avenues for therapeutic interventions (Li et al.). In addition, new colorectal cancer (CRC)-associated species were found, such as *Porphyromonas endodontalis*, *Ruminococcus torques*, and *Odoribacter splanchnicus*. Additionally, certain stage-specific bacterial taxa were identified, which may aid in diagnosing colorectal polyps (BP) and the four distinct CRC stages (Cai et al.). Furthermore, specific bacterial species and metabolic pathways may influence the occurrence of a series of immune-related adverse events (irAEs) in gastric, esophageal, and colon cancers. Notably, *Ruminococcus callidus* and *Bacteroides xylanisolvens* were enriched in patients without severe irAEs. Several microbial metabolic pathways, including citrulline and arginine biosynthesis, were associated with irAE development (Zhang et al.).

## Single-cell analysis and spatial resolution of the gut microbiome

The authors reviewed innovative microbial single cell sequencing techniques, highlighting their broad applications in addressing critical questions regarding microbiome composition and spatial heterogeneity. These advancements offer deeper insights into the functional roles and interactions of individual microbial cells within complex communities (Madhu et al.).

## Microbiome and neurologic disorders

The microbiota-gut-brain axis plays a pivotal role in regulating neuroprotective functions in the host. Patients with Parkinson’s disease (PD) often exhibit gut microbiota dysbiosis (Jia et al.). Moreover, the gut microbiome and its associated metabolic pathways appear to play a significant role in the long-term development of depression (Li et al.). In addition, the identification of bacterial classes like Negativicutes and Selenomonadales provides a new direction for therapeutic strategies to manage sleep disorders via microbiota modulation (Wang et al.). In another study, ten gut microbiome (GM) taxa were found to have causal associations with insomnia (Li et al.).

## Microbiome and women’s health

### Oral microbiome changes associated with the menstrual cycle

During the follicular phase, the relative abundance of the Streptococcus genus was significantly higher compared to the early and late luteal phases, while the Prevotella 7 and Prevotella 6 genera exhibited significantly lower abundance during the follicular phase than in the luteal phases. Alpha diversity was notably lower in the follicular phase, and beta diversity analyses revealed distinct microbial profiles across the four menstrual phases (Yamazaki et al.).

### Use of probiotic lactobacilli in the treatment of vaginal infections

Probiotic Lactobacilli play a crucial role in maintaining the balance of the vaginal microenvironment. A review explores their therapeutic potential in treating female vaginal infections. Probiotic lactobacilli contribute to restoring and preserving vaginal health by competing with pathogenic bacteria, enhancing the local immune response, and producing antimicrobial substances (Liu et al.).

### The urinary microbiota signatures and urinary diversion

The urinary microbiota signatures associated with different types of urinary diversion was studied. Urinary microbial landscapes of radical cystectomy and urinary diversion (UD) patients were analyzed (Liu et al.).

## Microbiome and respiratory infection

### Sputum microbiota characteristics and severe and critically ill influenza patients

*Bacteroidetes* showed significant depletion in the critically ill cohort. The sputum microbiomes in the severe influenza group were marked by an overrepresentation of *Neisseria*, *Porphyromonas*, *Actinobacillus*, *Alloprevotella*, *Nanosynbacter lyticus* TM7x, and *Clostridia* UCG-014. Notably, *Alloprevotella* exhibited an inverse correlation with *influenza* cycle threshold (Ct) values. Additionally, C-reactive protein (CRP) levels demonstrated a positive correlation with the presence of *Haemophilus* and *Porphyromonas* (Gu et al.).

## Microbiome and *Clostridioides difficile* infection and split-dose bowel preparations

### The gut microbiome and *Clostridioides difficile* infection

Distinct microbiome patterns were identified among healthy individuals, colonized patients, those with *Clostridioides difficile* infection (CDI), recurrent *Clostridioides difficile* infection (R-CDI), and patients with non-*Clostridioides difficile* infection (NOCDI) diarrhea. Potential microbiome biomarkers were discovered that may be valuable in distinguishing true CDI infections from other conditions, improving diagnostic accuracy and guiding treatment strategies (Vázquez-Cuesta et al.).

### Gut microbiota in children with split-dose bowel preparations

In pediatric patients undergoing split-dose PEG bowel preparation and colonoscopy, gut microbiota showed significant alterations at the genus, species, and functional pathway levels. However, no significant changes were observed at the phylum level (Zou et al.).

### Microbiome in fatty liver disease, gallstones, and cholesterol metabolism

Correlations between specific oral and gut fungal species with clinical parameters were identified from patients with Metabolic Dysfunction-Associated Fatty Liver Disease (MAFLD) patients (Niu et al.). One study reveals significant differences in microbial profiles between cholesterol and pigment gallstone patients (Zhang et al.).

## Disinfectants for pathogenic fungi

A combination of NaClO and H₂O₂ has shown potential as a more effective disinfectant, particularly against fungal pathogens, offering an alternative solution for more efficient microbial control (Li et al.).

## Conclusion

In conclusion, the integration of meta-omics techniques has revolutionized our ability to explore the microbiome’s contributions to human diseases, providing a more detailed understanding of microbial functions and their impact on human health and diseases. As these techniques continue to evolve, they hold immense promise for identifying novel diagnostic biomarkers and therapeutic targets across a wide range of diseases.
